# Chunking dynamics: heteroclinics in mind

**DOI:** 10.3389/fncom.2014.00022

**Published:** 2014-03-14

**Authors:** Mikhail I. Rabinovich, Pablo Varona, Irma Tristan, Valentin S. Afraimovich

**Affiliations:** ^1^BioCircuits Institute, University of CaliforniaSan Diego, La Jolla, CA, USA; ^2^Grupo de Neurocomputación Biológica, Departamento de Ingeniería Informática, Escuela Politécnica Superior, Universidad Autónoma de MadridMadrid, Spain; ^3^Instituto de Investigación en Comunicación Óptica, Universidad Autónoma de San Luis PotosíSan Luis Potosí, México

**Keywords:** cognitive dynamics, stable heteroclinic channel, transient dynamics, low dimensionality of brain activity, hierarchical sequences, chunking and superchunking, cognition modeling principles

## Abstract

Recent results of imaging technologies and non-linear dynamics make possible to relate the structure and dynamics of functional brain networks to different mental tasks and to build theoretical models for the description and prediction of cognitive activity. Such models are non-linear dynamical descriptions of the interaction of the core components—brain modes—participating in a specific mental function. The dynamical images of different mental processes depend on their temporal features. The dynamics of many cognitive functions are transient. They are often observed as a chain of sequentially changing metastable states. A stable heteroclinic channel (SHC) consisting of a chain of saddles—metastable states—connected by unstable separatrices is a mathematical image for robust transients. In this paper we focus on hierarchical chunking dynamics that can represent several forms of transient cognitive activity. Chunking is a dynamical phenomenon that nature uses to perform information processing of long sequences by dividing them in shorter information items. Chunking, for example, makes more efficient the use of short-term memory by breaking up long strings of information (like in language where one can see the separation of a novel on chapters, paragraphs, sentences, and finally words). Chunking is important in many processes of perception, learning, and cognition in humans and animals. Based on anatomical information about the hierarchical organization of functional brain networks, we propose a cognitive network architecture that hierarchically chunks and super-chunks switching sequences of metastable states produced by winnerless competitive heteroclinic dynamics.

## Introduction

Chunking is a dynamical phenomenon that the brain uses for processing long informational sequences. The concept of chunk was introduced by Miller (1956). His key notion is that short-term storage is not rigid but amenable to strategies such as chunking that can expand its capacity. Miller's work drew plenty of attention to the concept of short-term memory and its functional characteristics. Chunking involves two processes: concatenation of units in a block and segmentation of the blocks. In general, chunking is related to the hierarchical organization of perceptual, cognitive, or behavioral sequential activity. In particular, in motor control (see Rosenbaum et al., 1983) sequences can consist of sub-sequences and these can in turn consist of sub-sub-sequences, etc. The natural hierarchical organization of long sequences is a result of the activity of specific brain functional networks. Such networks include many different brain areas and some of them are also organized in a hierarchical manner. A well-known example is Broca's area that has been suggested to act as a “supramodal syntactic processor,” able to process any type of hierarchically organized sequences (Grossman, 1980; Tettamanti and Weniger, 2006), a hypothesis based on the findings that this region is not only involved in processing language syntax (Musso et al., 2003), but also in syntax like aspects of non-linguistic tasks, for example, the performance of specific movements and music (Fadiga et al., 2009) as several fMRI studies (Bahlmann et al., 2008, 2009) seem to confirm. Clerget et al. hypothesize that motor behavior shares some similarities with language (Clerget et al., 2013), namely that a complex action can be viewed as a chain of subordinate movements, which need to be combined according to certain rules in order to reach a given goal (Dehaene and Changeux, 1997; Dominey et al., 2003; Botvinick, 2008).

What are the mechanisms that transform the extremely complex, noisy, and many-dimensional brain activity into a rather regular, low-dimensional, and even predictable cognitive behavior, e.g., what are the mechanisms underlying the dynamics of the mind, including chunking? This is one of the most challenging questions in today's neuro- and cognitive science. Recent continuous advances in non-invasive brain imaging allow assessing the structural connectivity of the brain and the corresponding evolution of the spatio-temporal activity in detail.

In our view, metastability is a key element of transient cognitive dynamics participating in chunking processes. The idea of the spatiotemporal organization of brain dynamic activity through transient, metastable states emerged more than 15 years ago (Kelso, 1995; Friston, 1997). According to this scenario, such dynamics can be represented as a sequential switching between different metastable states (for a description of the mathematical basis of this scenario see Rabinovich et al., 2008a,b). Metastable transient dynamics represent a balance between the segregation of focused cognitive processing and the flexible integration of distributed brain areas. Such integration is necessary for the performance of a specific cognitive function (Bressler and Kelso, 2001; Meehan and Bressler, 2012). The existence of connections that are prevalent over long periods of time supports the well-regarded concept of a hierarchical organization of neural processing (Engel et al., 2001), which is the basis for the understanding of the origin of the chunking dynamics. Because the dimensionality of cognition depends on the number of activated (in contrast to the potentially observable) metastable states, it is important to remember that the brain chooses the necessary metastable states and suppresses those which are irrelevant to the goal of the cognitive process, resulting in a reduced dimensionality. The low-dimensionality of brain cognitive dynamics is based on two important issues: first, the manner of the cognitive task encoding—an external or internal stimulus determining a specific cognitive task excites a set of elements of the community networks which are responsible for the performance of such cognitive activities; and second, the existence of a specific hierarchical organization of the global brain networks that operate for the performance of a specific cognitive task by a moderate number of brain modes.

Based on experimental data suggesting that the processing of sequential cognitive activity on computational grounds is implemented in the brain by spatiotemporally pattern dynamics (see also Sahin et al., 2009), we build here a general dynamical model that produces hierarchical chunking of sequences, which suggests a plausible neural mechanism of chunking dynamics in the brain. This model is reasonably low-dimensional, which allows a detailed dynamical analysis.

## Materials and methods

A top-down approach to model transient cognitive dynamics taking into account the experimental observations described in the introduction is to use kinetic equations for the description of spatiotemporal mental modes that contain the discussed metastable states as equilibrium points. The set of brain patterns that sequentially change in the process of the cognitive task performance determine the spatial structure of the modes and the associated connection matrix among them. Using such type of models we can integrate our knowledge about the description of brain activity based on these new ideas related to heteroclinic sequences and their interactions, i.e., heteroclinic networks.

As a top-down departing point, we need a mathematical object that can describe robust transient dynamics and their associated information processing. Once we have this object, we can implement it through a set of canonic equations that can be used to study transient activity at different brain description levels, and in particular to address chunking dynamics. A mathematical image of robust transient sequential dynamics must have two principal features. First, it must be resistant to noise and reliable even in the context of small variations in initial conditions, so that the succession of states visited by the system (its trajectory, or transient) is stable. Second, the transients must be input-specific to contain information about what caused them. These are two fundamental contradictions regarding the use of transient dynamics for the description of brain activity. Transient dynamics are inherently unstable: any transient depends on initial conditions and cannot be reproduced from arbitrary initial conditions. On the other hand, dynamical robustness in principle prevents sensitivity to informative perturbations. These contradictions can be solved through the concept of metastability, which was introduced to cognitive science at the end of the last century (Kelso, 1995; Friston, 1997, 2000; Fingelkurts and Fingelkurts, 2006; Oullier and Kelso, 2006; Gros, 2007; Ito et al., 2007).

A stable heteroclinic channel (SHC) is a mathematical object that meets the above discussed requirements, which can implement such stable transients. A SHC is defined by a sequence of successive metastable “saddle” states that are connected by separatrices. Under proper conditions, all the trajectories in the neighborhood of these saddle metastable states that form the chain remain in the channel, ensuring robustness and reproducibility over a wide range of control parameters (Rabinovich et al., 2008b). The stability of a channel means that trajectories in the channel do not leave it until the end of the channel is reached.

A simple model to implement SHCs is a generalized Lotka–Volterra equation with *N* interactive elements:

(1) $$\begin{array}{l} \frac{dA_{i}(t)}{dt}=A_{i}(t)F\left(\sigma_{i}\left(S_{k}\right)-\sum_{j=1}^{N}\rho_{ij}A_{i}(t)\right)+A_{i}(t)\eta_{i}(t)\\ \;i=1,\ldots ,N\; \end{array}$$

where *A*_i_(*t*) ≥ 0 is the activity rate of element *i*, σ_*i*_ is the gain function that controls the impact of the stimulus, *S*_*k*_ is an environmental stimulus, ρ_*ij*_ determines the interaction between the variables, η_*i*_ represents the noise level, and *F* is a function, in the simplest case a linear function. The state portrait of the system often contains a heteroclinic sequence linking saddle points. These saddles can be interpreted as successive and temporary winners in a never-ending competitive game, i.e., winnerless competition (WLC) dynamics (Rabinovich et al., 2001, 2006). In neural systems, because a representative model must produce sequences of connected neuronal population states (the saddle points), the neural connectivity ρ_*ij*_ must be asymmetric, as determined by the theoretical examination of this model (Huerta and Rabinovich, 2004). Although many connection statistics probably work for stable heteroclinic-type dynamics, it is likely that connectivity within biological networks is, to some extent at least, the result of optimization by evolution and synaptic plasticity. It is important to emphasize that Equation (1) is just an elementary building block for different levels of the chunking hierarchy that we will describe below.

Models like the generalized Lotka–Volterra equations allow establishing the conditions necessary for transient stability, and display stable, sequential, and cyclic activation of its components, the simplest variant of WLC. A network with several degrees of freedom and asymmetric connections can generate structurally stable sequences—transients, each shaped by one input. Asymmetric inhibitory connectivity helps to solve the apparent paradox that sensitivity and reliability can coexist in a network (Huerta and Rabinovich, 2004; Nowotny and Rabinovich, 2007; Rabinovich et al., 2008b; Rabinovich and Varona, 2011). The neurons or modes participating in a SHC are assigned by the stimulus, by virtue of their direct and/or indirect input from the neurons activated by that stimulus. The joint action of the external input and a stimulus-dependent connectivity matrix defines the stimulus-specific heteroclinic channel. In addition, asymmetric inhibition coordinates the sequential activity and keeps a heteroclinic channel stable.

The WLC concept is directly related to the sequential dynamics of metastable states that are activated by inputs that do not destroy the origin of a competitive process. This paradigm can explain and predict many dynamical phenomena in neural networks with excitatory and inhibitory synaptic connections. Based on the requirement of the stability, this formalism has been used (i) to assess the dynamical origin of finite working memory (WM) capacity based upon WLC amongst available informational items (Bick and Rabinovich, 2009; Rabinovich et al., 2012); (ii) to build a dynamical model of information binding for transients that can describe the interaction of different sensory information flows that are generated concurrently (Rabinovich et al., 2010a); (iii) to model the sequential interaction between emotion and cognition (Rabinovich et al., 2010b); (iv) to represent attention dynamics (Rabinovich et al., 2013); and (v) to assess the dynamics of pathological states in mental disorders (Bystritsky et al., 2012; Rabinovich et al., 2013). Here we focus on a model of hierarchical chunking dynamics that can represent several forms of cognitive activity such as WM and speech construction.

As we discussed in the Introduction, chunking is grouping or categorizing related issues or information into smaller, most meaningful and compact units. Think about how hard it would be to read a long review paper without chapters, subchapters, paragraphs, and separated sentences. Chunking is a naturally occurring process that can be actively used to break down problems in order to think, understand, and make improvisation more efficiently. This is because it is easier to process chunked tasks or perceptional data. In particular, it is much easier to learn and recall such data. Mathematically, the “chunking principle” can be viewed as the transformation of a chain of metastable states along a transient process to the chain of groups of such states. It is a key dynamical idea that nature may use to make cognitive information processing more effective in the context of a complex environment.

Chunking processes in human perception, learning, and performance of a cognitive task can be both automatic and directly linked to the environmental stimuli, and controllable by a goal-oriented intrinsic signal (Gobet et al., 2001). It is important to note that chunking is a strategy that supports increasing speed and accuracy through the formation of hierarchical memory structures and complex task-dependent behavioral sequences. Two competitive processes form temporal chunking sequences—one separates long sequences into shorter groups of information items to be easily performed, and the second connects them to express a long sequence as a unified thought or behavioral action (Friederici et al., 2011; Chekaf and Matha, 2012).

**Figure 1:**
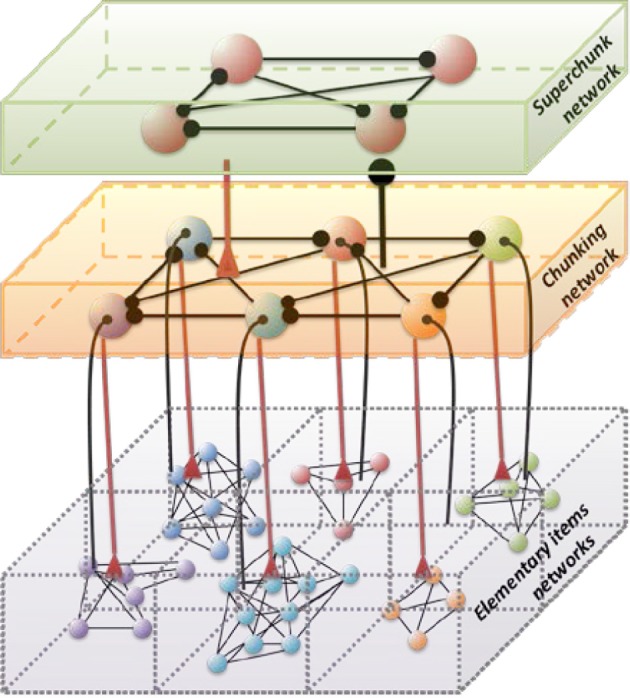
**Architecture of the three level cognitive network responsible for the grouping of informational items**. Each level of hierarchy is described by its own Lotka–Volterra type Equations (see 2–6) with connection matrices **ρ**, **ξ** and **ς**. Black circles represent inhibitory connections; triangles represent excitatory connections responsible for the choosing of the informational items. Spheres represent the informational items or units (metastable stables). Different colors indicate different chunks. All connections inside the elementary items are inhibitory.

Hierarchical chunking dynamics can be implemented in a model of cognitive networks whose information processing relies on SHCs. Figure 1 illustrates a chunking heteroclinic cognitive network for two hierarchical informational groups—elementary items and chunking (integrated) informational items including many elementary units interacting through dynamical connections. It is reasonable to hypothesize that functionally there are two different cognitive networks from at least two different hierarchical levels that are responsible for the: (i) organization of the sequence of items inside chunks, and (ii) the formation of the chunk sequence. In particular, this hypothesis is supported by an experiment with chunking during visuomotor sequence learning (Sakai et al., 2003). It has been shown that each motor cluster is processed as a single memory unit—a chunk. A learned visuomotor sequence is a sequence of chunks that contains several elementary movements. The authors of this work have shown that a key role in the process of chunking formation is played by a brain network including the dominant parietal area, the basal ganglia, and the presupplementary motor area (see also Ribas-Fernandes et al., 2011 and Bor and Seth, 2012, where authors discuss the chunking structure of conscious processes).

Below we suggest a three level hierarchical model for the description of the chunking dynamics. Inhibition plays a key role in this model as is responsible for the execution of three functions: (i) competition between elementary informational items in order to produce stable sequences of metastable states, (ii) generation of the chunking sequence, and (iii) control of the performance of the sequential task. In recent years, the investigation of the hierarchical control between different levels of representation and information processing has become one of the hot subjects in cognitive science. This issue is important for understanding how the mind controls behavior and itself. In particular, the relationship between chunking (a sequence-level process) and task-set inhibition (a task-level process) in the performance of task sequences was investigated in (Koch et al., 2006; Schneider, 2007; Li et al., 2010), for a description of “chunks of chunks”—“superchunks” see Rosenberg and Feigenson (2013).

To understand the emergence of hierarchical chunking dynamics in a model we need to depart from Equation (1) in the following direction, c.f. Figure 1):

(2) $${\dot{X}}_{i}^{lk}=X_{i}^{lk}\left(\sigma_{i}^{lk}(S,C)\cdot Y^{lk}-\sum_{j}^{N^{lk}}\rho_{ij}^{lk}(S,C)X_{j}^{lk}\right)$$

(3) $$\tau {\dot{Y}}^{lk}=Y^{lk}\left(\left(V^{l}-\beta (C)\sum_{i}^{N^{lk}}X_{i}^{lk}\right)-Z^{lk}\right)$$

(4) $$\theta (C){\dot{Z}}^{lk}=\sum_{m}^{M}\xi_{l}^{km}(S,C)Y^{lm}-Z^{lk}$$

(5) $$T{\dot{V}}^{l}=V^{l}\left(\left(1-\delta (C)\sum_{j}^{M^{l}}Y^{lj}\right)-W^{l}\right)$$

(6) $$\Theta (C){\dot{W}}^{l}=\sum_{q}^{P}\varsigma^{lq}(S,C)V^{q}-W^{l}$$

Here *X^lk^_i_* characterizes the -th informational item associated with the *k*-th chunk and *l*-th superchunk, σ*^lk^_i_*(*S, C*) is the growth rate for each informational item determined by the stimulus *S* and the cognitive task *C*, and ρ*^lk^_ij_*(*S, C*) is the matrix of inhibitory connections among basic informational items. In this model *Y*^*lk*^ characterizes the *k*-th chunk associated to the *l*-th superchunk *V*^*l*^, with corresponding characteristic times τ and *T*, respectively, and β(*C*) represents the strength of the inhibition between the informational items and the chunk, and δ(*C*) between the chunks and the superchunk. Also, *Z*^*lk*^ describes the synaptic dynamics for the *k*-th chunk associated to the *l*-th superchunk with ξ*^km^_l_*(*S, C*), the matrix of inhibitory connections between chunks (black circles in Figure 1); and *W*^*l*^ describes the synaptic dynamics for the *l*-th superchunk with **ς**^*lq*^(*S, C*), the matrix of inhibitory connections between superchunks, the corresponding characteristic times are θ(*C*) and Θ(*C*). In this model, β(*C*) and δ(*C*) are adaptation parameters that determine the timing relationship between a basic informational chain and the chunking and superchunking modulation. The chunking variables also satisfy the generalized Lotka–Volterra—canonic equations which allows them to form a stable sequence. Because of this, in fact, chunking variables play the role of cognitive controllers. The parameters for Equations (3)–(5) in the simulations below were chosen with this scope. Since chunking dynamics has to take into account of the characteristic time of the chunk formation, the competition between different chunks has to be delayed—we used for this an inhibition described by a first order kinetic model. At the same time, the competition among elementary informational items is implemented by fixed weight ρ_*ij*_ instantaneous synapses. The same logic has been applied for the description of the highest level of the hierarchy—the superchunks.

## Results: hierarchical sequences—chunking and super-chunking

**Figure 2:**
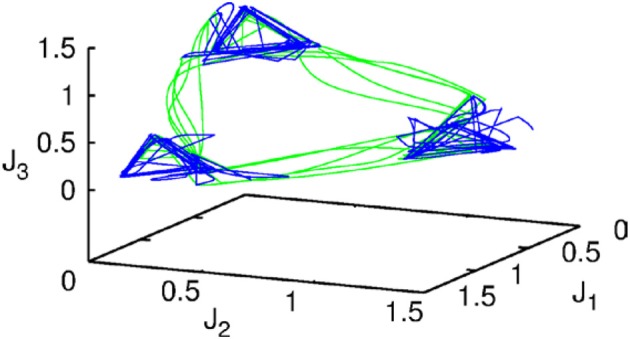
**The projection of a nine-dimensional phase portrait of a two-level chunking hierarchical dynamics in the space of the three-dimensional auxiliary variables [see the Equations (2)–(4)] *J*_1_ = *Y*^1^ + 0.04 · (*X*^1^_1_ + *X*^2^_1_ + *X*^3^_1_), *J*_2_ = *Y*^2^ + 0.04 · (*X*^1^_2_ + *X*^2^_2_ + *X*^3^_2_), *J*_3_ = *Y*^3^ + 0.04 · (*X*^1^_3_ + *X*^2^_3_ + *X*^3^_3_)**. Blue represents the elementary informational item activity—individual chunk. Green represents the chunking sequence.

**Figure 3:**
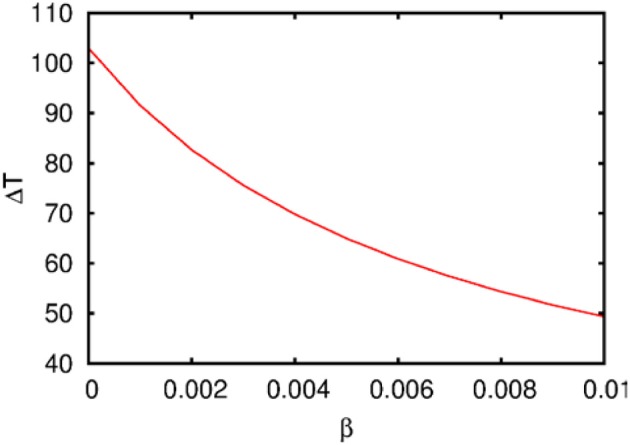
**The dependence of the chunking interval timing [see Equation (1)] on the control parameter β**. *One* can see that the chunking interval strongly decreases together with the increasing of the adaptation parameter β. When β increases the effective excitation of variable *Y* decreases.

Let us first represent the phase portrait of a simple two-level chunking dynamics. We carried out numerical simulations of the model for the dynamics within chunks of informational items for the following parameters *N*^k^ = 3, *M* = 3 (number of “chunks” or “episodes”), σ ^1^ = [7.24, 5.85, 8.30], σ ^2^ = [9.93, 6.00, 5.18], σ ^3^ = [8.29, 7.86, 9.16], and given these values, $\rho_{ii}^{k}=1.0,\rho_{i_{n-i}i_{n}}^{k}=\frac{\sigma_{i_{n-1}}^{k}}{\sigma_{i_{n}}^{k}}+0.51$, and $\rho_{i_{n+i}i_{n}}^{k}=\frac{\sigma_{i_{n+1}}^{k}}{\sigma_{i_{n}}^{k}}-0.5$, *i* = 1,…,*N^lk^*, *k* = 1,…,*M* as well as the parameters considered for the synaptic dynamics described by Equations (3) and (4): τ = 0.7, θ = 2.0, ξ^*kk*^ = 1.0, ξ^*k*_*n*_*k*_*n* + 1_^ = 1.4 and ξ^*k*_*n*_*k*_*n* − 1_^ = 0.5, *k* = 1,…,*M* and β = 0.01. The results of these simulations are shown in Figures 2, 3.

Figure 2 shows the phase portrait of the chunking dynamics when the superchunk formation is absent: the system is described by Equations (2)–(4), *V* = 1. This example illustrates a closed chunking sequence (green) that consists of several heteroclinic cycles that represent the elementary chunks (blue). In general, the number of elementary items in each chunk are different and the chunking sequence can be open.

Figure 3 illustrates the timing between chunks along the sequence. The emergence of the chunking sequence shown in Figure 2 is the result of a modulational instability in the two-level hierarchical network whose dynamics is described by Equations (2)–(4). This instability is oscillatory. The characteristic period of the oscillation is ΔT. The analytical investigation of the dependence of ΔT on the control parameters τ, θ, β and connection matrices **ρ**, **ξ** is a non-realistic problem because of the non-linear feedback between the dynamical variables *X* and *Y*. However, it is reasonable to think that the key parameter in this problem is β which determines the level of excitability of variable *Y* and, according to the feedback, also controls the excitability of *X* (term σ*^lk^_i_*(*S, C*) · *X^lk^_i_* · *Y^lk^*) in the right hand side of Equation (2). In Figure 3 we represent the numerical analysis of the dependence of ΔT on the parameter β —increasing β, i.e., decreasing the excitability leads to the decreasing of the timing interval ΔT.

**Figure 4:**
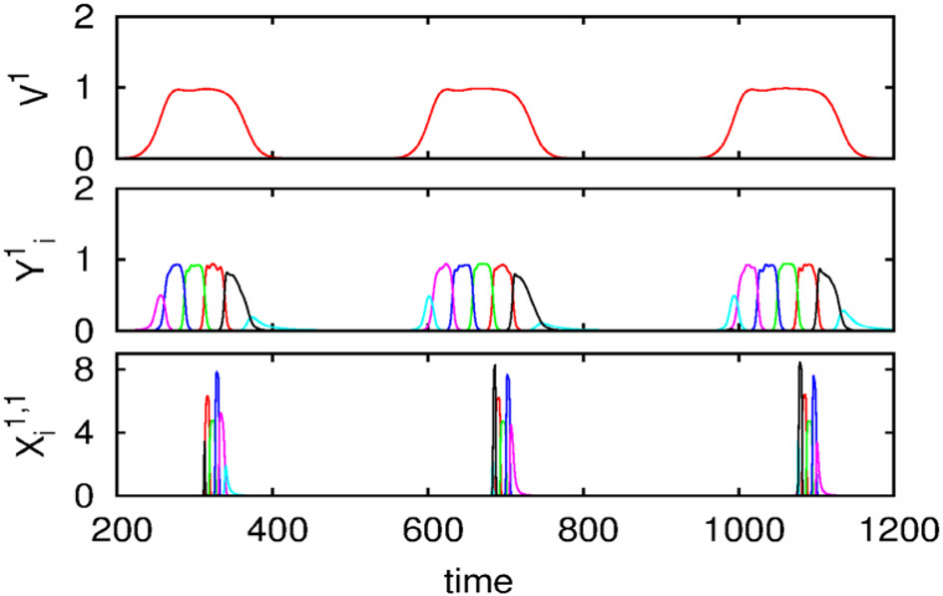
**Time series of the sequences of the three-level hierarchy—108 items groupped in 18 chunks of 6 items; these chunks form 3 superchunks of 6 elements each displaying reproducible dynamics according to the model (2)–(6)**. Different colors correspond to different items inside each group (switching the color means moving from the previous item to the next one).

We also carried out numerical simulations of a high-dimensional model that describes the dynamics of chunk and super-chunk formation with the following parameters: *N*^*lk*^ = 6, *M*^*l*^ = 6 (number of chunks), *P* = 3 (number of superchunks), σ^*l*1^ = [6.94, 5.11, 8.94, 5.86, 8.33, 9.62], σ^*l*2^ = [5.48, 5.66, 5.39, 9.89, 9.99, 5.82], σ ^*l*3^ = [7.65, 8.98, 9.21, 6.02, 5.71, 5.12], σ ^*l*4^ = [7.61, 7.73, 5.62, 7.93, 5.80, 5.39], σ ^*l*5^ = [5.11, 9.99, 5.52, 5.66, 5.50, 8.21], σ ^*l*6^ = [5.84, 9.39, 7.08, 5.16, 8.37, 6.87], and given these values, $\rho_{i_{n-i}i_{n}}^{lk}=\frac{\sigma_{i_{n-1}}^{lk}}{\sigma_{i_{n}}^{lk}}+0.5$ 1, $\rho_{i_{n+i}i_{n}}^{lk}=\frac{\sigma_{i_{n+1}}^{lk}}{\sigma_{i_{n}}^{lk}}-0.5$, *i* = 1,…,*N^lk^*, *k* = 1,…,*M^l^*, *l* = 1,…,*P*, and $\rho_{ii_{n}}^{lk}=\rho_{i_{n-1}i_{n}}^{lk}+\frac{\sigma_{i}^{lk}-\sigma_{i_{n-1}}^{lk}}{\sigma_{i_{n}}^{lk}}+2,$, *i* ≠ {*i*_*n* − 1_, *i*_*n*_, *i*_*n* + 1_}, as well as the parameters considered for the synaptic dynamics between chunks described by the equations τ = 0.8, θ = 2.0, ξ*^kk^_l_* = 1.0, ξ^*k*_*n*_*k*_*n* − 1_^_l_ = 0.5, ξ^*k*_*n*_*k*_*n* + 1_^_1_ = 1.4, ξ^*k*_*n*_*k*_*n* + 1_^_2_ = 1.3, ξ^*k*_*n*_*k*_*n* + 1_^_3_ = 1.5, *k* = 1,…,*M*^*l*^, *l* = 1,…,*P*, ξ^*kk*_*n*_^_l_ = ξ^*k*_*n* − 1_*k*_*n*_^_l_ + 2, *k* ≠ {*k*_*n* − 1_, *k*_*n*_, *k*_*n* + 1_}, and β = 0.01. Finally, the parameters for the synaptic dynamics between superchunks were *T* = 5, Θ = 10, ς^*ll*^ = 1.0, ς^*l*_*n*_*l*_*n* − 1_^ = 0.5, ς^*l*_*n*_*l*_*n* + 1_^ = 1.4, *l* = 1,…,*P*, and δ = 0.01. The result of these simulations are displayed in Figure 4, which shows three levels of information hierarchy: original informational chain (lower panel), chunked chain (middle panel), and superchunking chain (upper panel).

As illustrated in Figure 2, the sequence of chunks can be considered as a heteroclinic cycle of metastable states where each metastable state itself is a heteroclinic cycle of elementary informational items. Based on this self-similarity, we can expect that the chunking chain as a result of a second heteroclinic instability generates the next level of modulation—the superchunk sequence. Our expectation is confirmed in Figure 4 that shows the time series of the three level network (2)–(6) (c.f. Figure 1) dynamics. In this figure, one can see the generation of sequences of superchunks. All together, the sequences informational items, chunks and superchunks can be interpreted as “words,” “sentences,” and “paragraphs.”

For the sake of simplicity we have illustrated here the phenomenon of stability just for a closed-loop clustered chunking-superchunking sequence. In the general case of open sequence, it is possible to formulate the sufficient conditions for the existence and stability of the non-closed channel based on the estimation of the saddle values of the metastable states (elementary items)—the channel is stable in the case that all of them are larger than one in absolute value (Afraimovich et al., 2004; Bick and Rabinovich, 2010). The formulation of the necessary conditions is a more complex problem and is still under consideration. The imposed stability conditions determine the behavior of the trajectories inside the neighborhood of the heteroclinic network independently of the initial conditions as computer experiments have confirmed (Afraimovich et al., 2004; Bick and Rabinovich, 2010).

The above described numerical results can be justified by an analytical study of the system

(7) $$\{\begin{array}{l} \begin{array}{l} {\dot{X}}_{i}^{k}=X_{i}^{k}\left(\sigma_{i}^{k}\cdot Y^{k}-\sum_{j=1}^{N^{k}}\rho_{ij}^{k}X_{j}^{k}\right)\text{​},\\ \tau {\dot{Y}}^{k}=Y^{k}\left(1-\beta \sum_{i=1}^{N^{k}}X_{i}^{k}-Z^{k}\right)\text{​},\; \end{array}\\ \theta {\dot{Z}}^{k}=\sum_{m=1}^{M}\xi^{km}Y^{m}-Z^{k} \end{array}$$

*i* = 1,…,*N^k^*, *k* = 1,…,*M*. For the sake of simplicity, let us assume that τ = θ << 1, so one can apply geometric singular perturbation theory (see, for instance, Jones, 1995; Hek, 2010 and references therein). In order to avoid confusion, it is important to say that the assumption τ = θ << 1 implies that, in contrast to the dynamics of *X*, the chunking dynamics is a composition of fast and slow motions. The fast motions lead variables *Y*-th and *Z*-th to a neighborhood of the slow manifold in the phase space. The evolution of the chunk variables on this manifold in the vicinity of the metastable states is much slower than the *X* variables. This corresponds to the intuitively clear fact that the “enveloping” variables mimic the averaging dynamics of *X*. Computer experiments confirm this explanation (see Figure 4).

The limit slow manifold has the equations $Y^{k}\left(1-\beta \sum_{i=1}^{N^{k}}X_{i}^{k}-Z^{k}\right)=0,\sum_{m=1}^{M}\xi^{km}Y^{m}-Z^{k}=0$, thus, $\sum_{m=1}^{M}\xi^{km}Y^{m}=\;1-\beta \sum_{i=1}^{N^{k}}X_{i}^{k}.$ Denote by ξ the *m* × *m*-matrix ξ^*km*^. If det ξ ≠ 0, we find

(8) $$Y^{k}=\frac{1}{\det\xi }\left(\sum_{m=1}^{M}\eta^{mk}-\beta \sum_{m=1}^{M}\eta^{mk}\sum_{i=1}^{N^{m}}X_{i}^{m}\right)$$

where η^*km*^ is the cofactor of the entry ξ^*mk*^ of the matrix ξ. Substituting this expression into the first equation of the system (7) we obtain the system

(9) $${\dot{X}}_{i}^{k}=X_{i}^{k}\text{​}\left(\sigma_{i}^{k}\frac{1}{\det\xi }\sum_{m=1}^{M}\eta^{mk}-\text{​}\sum_{j=1}^{N^{k}}\rho_{ij}^{k}X_{j}^{k}-\text{​}\frac{\beta }{\det\xi }\sum_{m=1}^{M}\eta^{mk}\sum_{i=1}^{N^{k}}X_{i}^{m}\right)$$

*i* = 1,…,*N^k^*, *k* = 1,…,*M*, which is similar to the binding model described in Rabinovich et al. (2010a). In particular, the “in-chunk” dynamics in (9) corresponds to the dynamics in the modality subspace in Rabinovich et al. (2010a). The main peculiarity of the system (9) is that the rates of coupling coefficients between different chunks have the common factor β, so if β = 0 then the interaction between different chunks is absent. Similarly to the study in Rabinovich et al. (2010a), one can impose conditions under which there exists a heteroclinic cycle for each chunk and successive heteroclinic connections between saddle points in different cycles. The last claim has the form β > β_*cr*_ where β_*cr*_ depends on the parameters of the system (9). If τ is small then because of the geometric singular perturbation theory, the imposed conditions shall guarantee the existence of a heteroclinic network in the original system (7) corresponding to the “in-chunk” and “inter-chunk” dynamics.

Observations on the temporal chunk signal have focused on the use of pauses in behavior to probe chunk structures in WM. On the basis of some of these studies, a hierarchical process model has been proposed, which consists of four hierarchical levels describing different kind of pauses. The lowest level consists of pauses between strokes within letters. On higher levels, there are pauses between letters, words, and phrases. Each level is associated with a larger amount of processing when retrieving these chunks from memory (Cheng and Rojas-Anaya, 2006). Writing may be an effective approach to the study of cognitive phenomena that involves the processing of chunks. In Cheng and Rojas-Anaya (2003), it was demonstrated that in the writing of simple number sequences the duration of pauses between written elements (digits) that are within a chunk are shorter than the pauses between elements across the boundary of chunks. This temporal signal is apparent in un-aggregated data for individual participants in single trials. Mathematically the time intervals between chunks and super-chunks are controlled by parameter β (see Equation 3).

## Discussion

In this paper we have shown how the architecture of hierarchical mental model networks affected their associated functions. The discussed examples illustrate that networks with metastable states having several unstable separatrices exhibit very diverse cognitive functions (behavior). Complex heteroclinic networks allow completely new dynamical phenomena, and one of the primary challenges is the assessment of the existence and stability of hierarchical—chunking processes that can represent cognitive activity.

It is important to remind that the modeling of cycling and sequential dynamics in behavior and cognition has a long history (see several representative efforts in Table 1). Most of these models are based on Hopfield type networks. The main problem there is to keep the stability of the recall sequences against noise.

**Table 1:** **Sequential dynamics in neural and cognitive systems**.

**Phenomenon/image**	**Model**	**References**	**Comments**
Voting paradox / Structurally stable heteroclinic cycle	Kinetic (rate) equation, Lotka–Volterra model	Krupa, 1997; Stone and Armbruster, 1999; Ashwin et al., 2003; Postlethwaite and Dawes, 2005	J. C. Borda and the Marquis de Condorcet (De Borda, 1781; Saari, 1995) analyzed the process of plurality elections at the French Royal Academy of Sciences. They predicted the absence of a winner in a 3 step voting process (Condorcet's triangle)
Learning sequences	Hopfield type non-symmetric networks with time delay including spiking neuron models	Amari, 1972; Kleinfeld, 1986; Sompolinsky and Kanter, 1986; Minai and Levy, 1993; Deco and Rolls, 2005	Networks proposed to explain the generation of rhythmic motor patterns and the recognition and recall of sequences
Latching dynamics	Potts network is able to hop from one discrete attractor to another under random perturbation to make a sequence	Treves, 2005; Russo et al., 2008; Russo and Treves, 2011; Linkerhand and Gros, 2013	The dynamics can involve sequences of continuously latching transient states
Sequential memory with synaptic dynamics / Chaotic itinerancy sequences of Milnor attractors or attractor ruins	Spike-frequency-adaptation mechanism Noisy dynamical systems. Cantor coding	Tsuda, 2009	Proposed to be involved in episodic memory and itinerant process of cognition
Winnerless sequential switchings along metastable states/Stable heteroclinic channel	Generalized coupled Lotka–Volterra equations	Afraimovich et al., 2004; Rabinovich et al., 2008a,b	Information processing with transient dynamics at many different description levels from simple networks to cognitive processes
Winnerless competitive dynamics in spiking brain networks	Random inhibitory networks of spiking neurons in the striatum	Ponzi and Wickens, 2010	Neurons form assemblies that fire in sequential coherent episodes and display complex identity–temporal spiking patterns even when cortical excitation is constant or fluctuating noisily
Sequences of sequences / Hierarchical transient sequences	Recognition of sequence of sequences based on a continuous dynamical model	Kiebel et al., 2009	Speech can be considered as a sequence of sequences and can be implemented robustly by a dynamical model based on Bayesian inference. recognition dynamics disclose inference at multiple time scales

The results of chunking dynamics reported in this paper can be viewed as relevant in the description of different cognitive tasks. For example, in WM, humans encode items and synthesize them. With that, we give meaning to ideas and find a relevant place for them in our cognitive world. In these actions the interaction between WM and chunking are reciprocal—first of all WM is the “engine” of chunking, and on the other hand, the chunking makes WM capacity higher.

The model of chunking dynamics discussed in this paper relies on heteroclinic dynamics. It is important to emphasize that the main features of the SHC do not depend on the specific model used. The conditions of existence and the dynamical features of SHCs can be implemented in a wide variety of models: from simple Lotka–Volterra descriptions to complex Hodgkin–Huxley models, and from small networks to large ensembles of many elements (Varona et al., 2002; Venaille et al., 2005; Nowotny and Rabinovich, 2007; Rabinovich et al., 2012). The intrinsic hierarchical nature of the SHC at different temporal and spatial scales allows implementing many types of cognitive dynamics. Within this framework, brain networks can be viewed as non-equilibrium systems and their associated computations as unique patterns of transient activity, controlled by incoming input. The results of these computations can be reproducible, robust against noise, and easily decoded. Using asymmetric inhibition appropriately, the space of possible states of large neural systems can be restricted to connected saddle points, forming SHCs. These channels can be thought of as underlying reliable transient brain dynamics. Table 2 summarizes four types of heteroclinic networks that can describe different aspects of sequential dynamics in cognitive processes: (i) A canonic heteroclinic network that produces reproducible sequential switching from one metastable state to another inside one modality (like in a simple WM task); (ii) A network displaying inhibitory-based heteroclinic binding dynamics that is responsible for the stable perception of a subject based on three different modalities; (iii) Two different modalities dynamically coordinated by excitatory connections; (iv) A chunking heteroclinic network that controls the grouping of elements of sequential behavior.

**Table 2:** **Heteroclinics in mind**.

**Phenomenon**	Network formalism^*^	**Phase portrait**	**Time series**
Sequential heteroclinic switching	${\dot{X}}_{i}=X_{i}\left(\sigma_{i}-\sum_{j=1}^{N}\rho_{ij}X_{j}\right)$	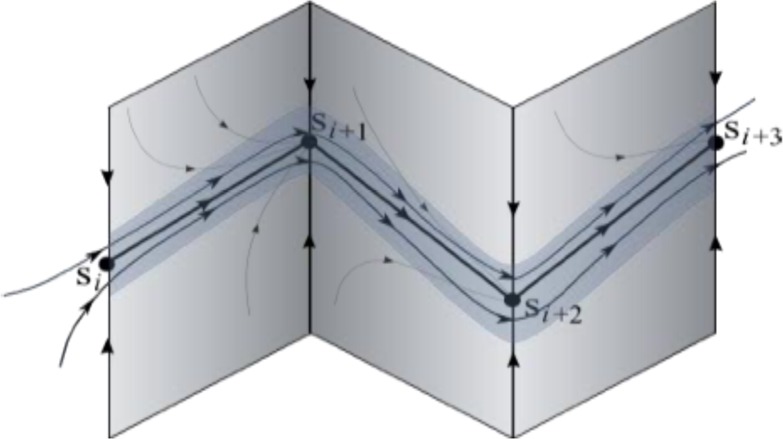	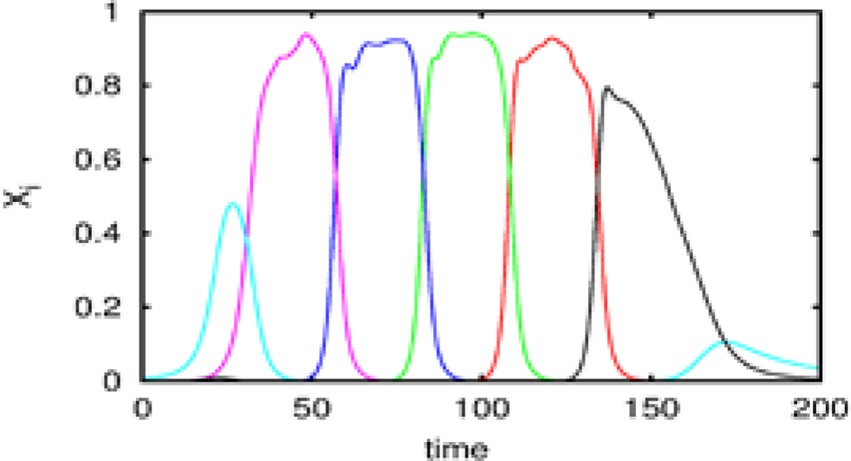
Sequential heteroclinic binding and information flow	${\dot{X}}_{i}^{l}=X_{i}^{l}\left(\sigma_{i}^{l}-\sum_{j=1}^{N}\rho_{ij}^{l}X_{j}^{l}-\sum_{m=1}^{L}\sum_{j=1}^{N}\xi_{ij}^{lm}X_{j}^{m}\right)$	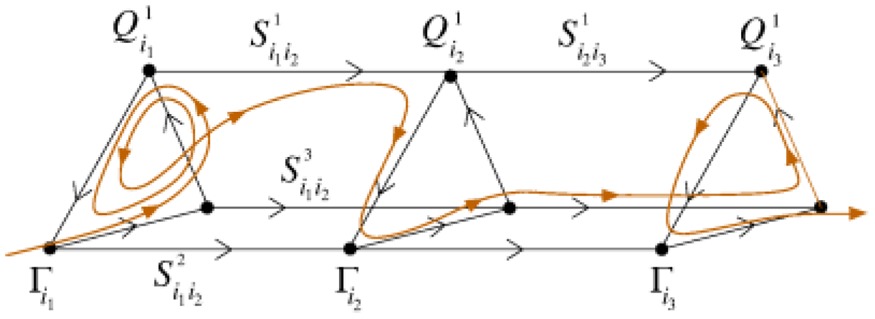	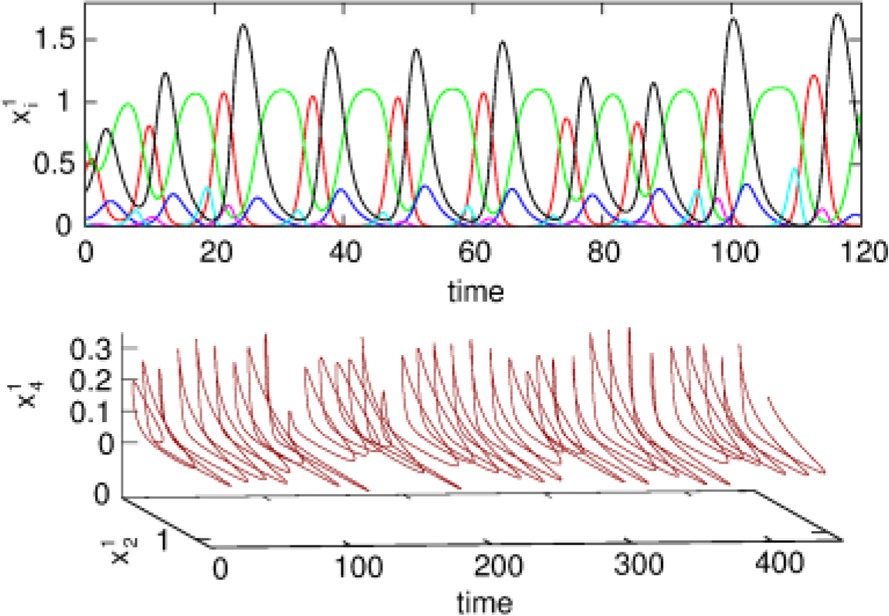
Heteroclinic cooperation	$\tau_{i}^{m}{\dot{X}}_{i}^{m}=X_{i}^{m}\cdot \left[\sigma_{i}^{m}-\sum_{j=1}^{K^{m}}\rho_{ij}^{m}X_{j}^{m}+\sum_{k=1}^{M}\sum_{j=1}^{K^{m}}\xi_{ij}^{mk}X_{j}^{k}\right]$	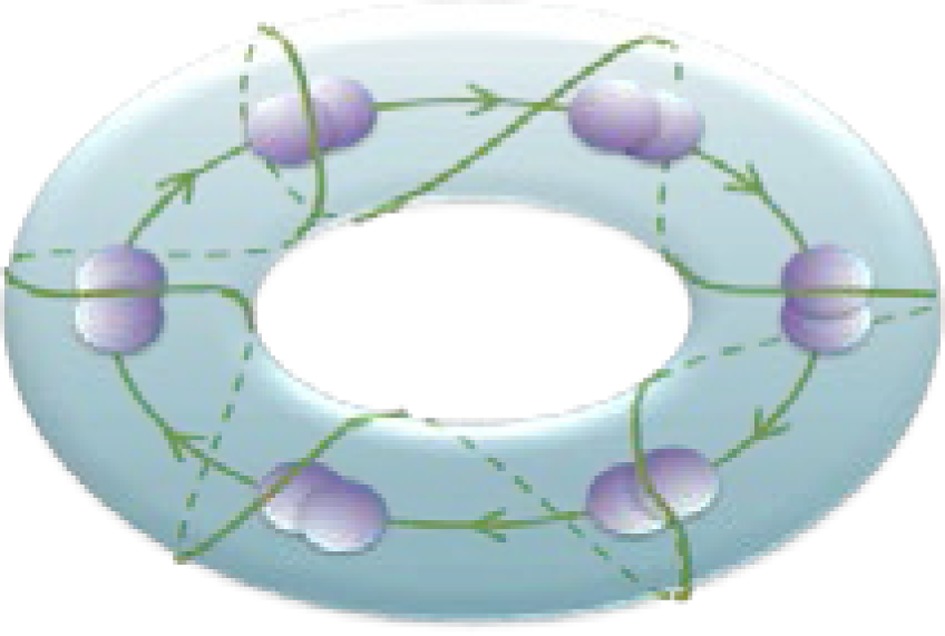	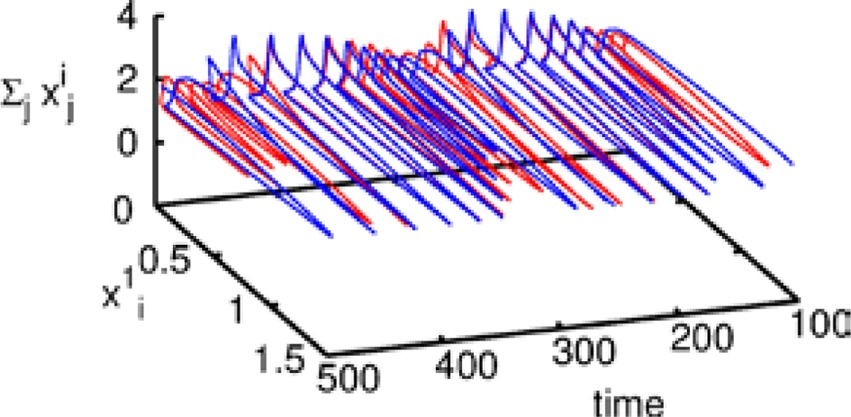
Hierarchical chunking memory and learning	$\begin{array}{l} {\dot{X}}_{i}^{k}=X_{i}^{k}\left(\sigma_{i}^{k}\cdot Y^{k}-\sum_{j}^{N^{k}}\rho_{ij}^{k}X_{j}^{k}\right)\\ \tau {\dot{Y}}^{k}=Y^{k}\left(\left(1-\beta \sum_{i}^{N^{k}}X_{i}^{k}\right)-Z^{k}\right)\\ \theta {\dot{Z}}^{k}=\sum_{m=1}^{M}\xi^{km}Y^{m}-Z^{k} \end{array}$	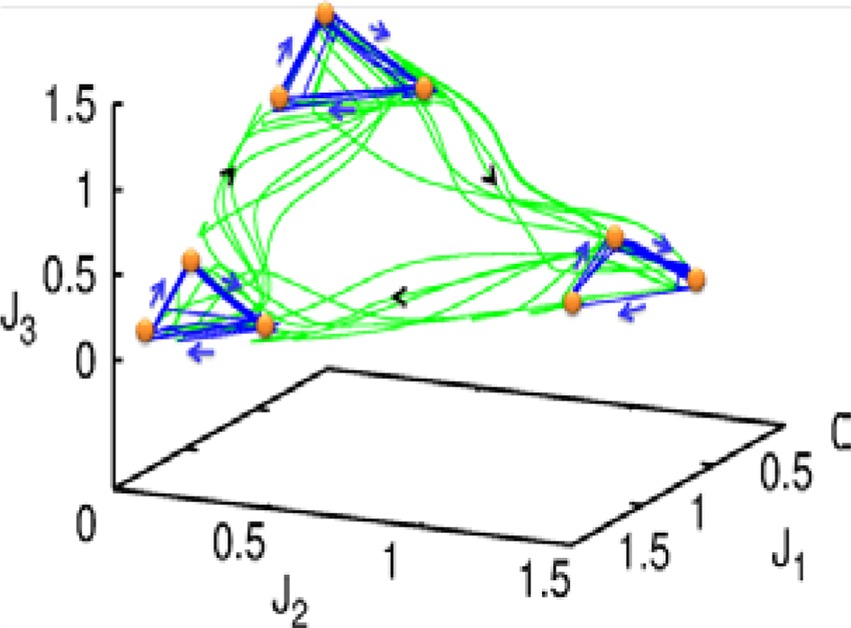	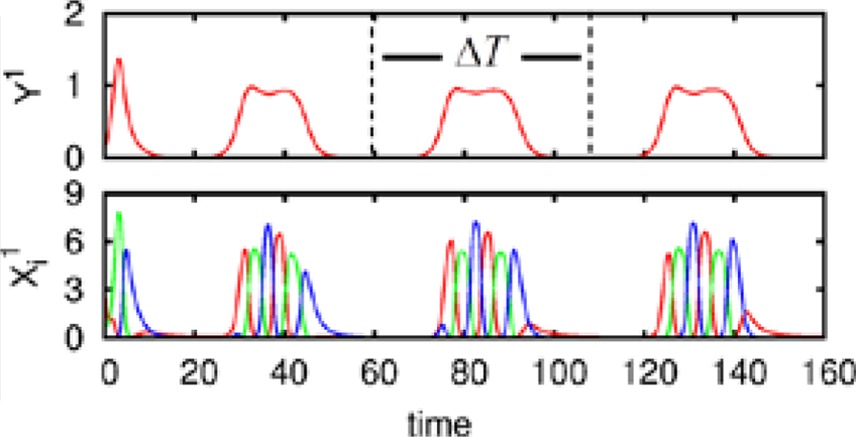

* *See the definition of the variables and parameters in the text*.

Mathy and Feldman have recently suggested to use the Kolmogorov complexity and compressibility (Mathy and Feldman, 2012) for the definition of a “chunk”: a chunk is a unit in a maximally compressed code. The authors presented a series of experiments in which they manipulated the compressibility of stimulus sequences by introducing sequential patterns of variable length. To explore the influence of chunking on the capacity limits of WM, and departing from Bick and Rabinovich (2009), authors in Li et al. (2013) have suggested a model for chunking in sequential WM. This model also uses hierarchical bidirectional inhibition-connected neural networks with WLC. Assuming no interaction between a basic sequence and a chunked sequence, and the existence of an upper bound to the inhibitory weights the network, authors show that chunking increases the number of memorized items in WM from the “magical number” 7–16 items. The optimal number of chunks and the number of the memorized items in each chunk correspond to the “magical number 4.”

Recent experiments have confirmed the existence of three levels of cognitive hierarchy—see Rosenberg and Feigenson (2013). In this paper authors reported that infants can unify the representation of chunks into *“super-chunks.”*

The chunking models discussed above can be generalized on more complex cases. In particular, by adding attention control in the network hierarchy, it is possible to analyze the binding of sequences of chunks. The brain could use such binding to perform many cognitive functions like the coordination of visual perception with speech comprehension, or the coordination of music chunks and word chunks in singing processes. It is well-known that viewing a speaker's articulatory movements substantially improves a listener's ability to understand spoken words, especially under noisy environmental conditions like in a crowded cocktail party. Ross and coauthors claimed that this effect is most pronounced when the auditory input is weakest. As a result of attentional binding—multisensory integration—, substantial gain in multisensory speech enhancement is achieved at even the lowest signal-to noise ratios (Ross et al., 2007).

The dynamics of hierarchical heteroclinic networks is also able to explain and predict the coordination of behavioral elements with different time scales (for a study about the coordination of sensorimotor dynamics see Jantzen and Kelso, 2007). Functionally, such kind of synchronization can be the result of learning—the changing of the strength of inhibitory connections between agents at the different levels of the hierarchy in order to coordinate the dynamics with different time scales (see Figure 3). Additionally, it is important to note that the winnerless competitive learning process itself can be chaotic (Komarov et al., 2010), which provides wider possibilities for adaptability.

### Conflict of interest statement

The authors declare that the research was conducted in the absence of any commercial or financial relationships that could be construed as a potential conflict of interest.
